# Applications and Innovations on Sensor-Enabled Wearable Devices

**DOI:** 10.3390/s22072599

**Published:** 2022-03-29

**Authors:** Joaquín Torres-Sospedra, Elena Simona Lohan, Antonella Molinaro, Adriano Moreira, Alexandru Rusu-Casandra, Zdenek Smékal

**Affiliations:** 1 ALGORITMI Research Centre, Universidade do Minho, 4800-058 Guimarães, Portugal; adriano.moreira@algoritmi.uminho.pt; 2 Electrical Engineering Unit, Tampere University, 33720 Tampere, Finland; 3 DIIES Department, University Mediterranea of Reggio Calabria, 89124 Reggio Calabria, Italy; antonella.molinaro@unirc.it; 4 Communications and Signal Processing Research Center, Telecommunications Department, Politehnica University of Bucharest, 060042 Bucuresti, Romania; alexandru.rusu@upb.ro; 5 Department of Telecommunications, Brno University of Technology, 616 00 Brno, Czech Republic; smekal@feec.vutbr.cz

## 1. Contributions to the Special Issue

Multiple sensors are embedded in wearable devices. Sensors are mainly included for tracking information on the user’s physical activity and physiological parameters, but additional sensors are also included for radio-communications and other purposes. This advanced complex sensory system enables wearables to be a source of invaluable crowdsourced data, where sensor fusion may enable innovative applications in many fields (engineering, telecommunications, computer science, eHealth, the Internet of Things, Sensor Networks, etc.).

The Special Issue on “Applications and Innovations on Sensor-Enabled Wearable Devices” in *Sensors* (MDPI) welcomed submissions of technological innovations and novel applications for wearable devices, with special interest in indoor positioning, from both Academia and the Industry.

A total of nine papers were published, contributing to the research community with the description of applications and reviews about the Special Issue’s topics. The published papers summed up a total of 63 citations in the Web of Science (WOS) records and 120 citations in the Google Scholar (GS) records at the moment of writing this editorial.

### 1.1. Proposed Applications

An automatic “museum audio guide” is presented in [1] as a new hands-free guide for museums. The proposed device is built as a headset equipped with a camera that captures exhibit pictures and a board that is capable of recognizing artworks using a random forest classifier based on the features from accelerated segment test keypoints. Two different use case scenarios and a pilot in a real museum setup were presented, showing the feasibility of the new proposed audioguide.

The work introduced in [2] proposed a solution for an objective assessment of two relevant motor symptoms of Parkinson’s Disease, namely, tremor and bradykinesia. Physical movements were recorded by means of a bracelet, i.e., the A-WEAR Bracelet, that included a 3D accelerometer and a gyroscope. Different machine learning models were assessed using the data collected with the A-WEAR bracelet, reaching an accuracy of 91.7% with the *k*-NN model. The data from A-WEAR bracelet have shown to be promising in order to detect presence of tremor and bradykinesia.

### 1.2. Systematic Reviews and Surveys

The systematic review introduced in [3] focuses on posture identification, activity recognition, and step counting for daily activity monitoring. In particular, the review is devoted to synthesizing the available information on the criterion validity of instrumented insoles in detecting posture activities. A total of 33 studies evaluated 17 different insole models, most of which were academic prototypes, and involved a total of 290 participants ranging from 16 to 75 years old. Criterion validity was assessed using six statistical indicators. Across studies, different postures and activities were assessed using different criterion validity indicators, leading to heterogeneous results. Instrumented insoles appeared to be highly accurate for steps counting. However, measurement properties were variable for posture and activity recognition. According to the authors, these findings call for a standardized methodology to investigate the measurement properties of such devices.

The work in [4] introduces a systematic analysis and introduces a novel fusion framework from an application perspective. The authors have identified that although densely deployed wearable sensors provide a platform for comprehensively monitoring the status of people, wearable technology based on multi-source fusion lacks a generalized research system to highlight the advantages of heterogeneous sensor networks and information fusion. Therefore, the authors propose a multi-level fusion framework (MLFF) based on Body Sensor Networks (BSNs) and describe a model of the deployment of heterogeneous sensor networks. The proposed framework covers multiple types of information at a single node, including behaviors, physiology, emotions, fatigue, environments, and locations, i.e., the proposed framework integrates low-level sensing data and high-level decision information to build a comprehensive and extensible application research system for Body Sensor Networks.

The systematic review introduced in [5] targets current and forthcoming wearable technologies, with a focus on sensing elements, body placement, detection accuracy, underlying algorithms, and applications in the context of monitoring cigarette smoking. A total of 86 scientific articles were reviewed in accordance with the Preferred Reporting Items for Systematic Review and Meta-Analyses (PRISMA) guidelines to address 3 research directions oriented to cigarette smoking: (1) Investigate the behavioral and physiological manifestations of cigarette smoking targeted by wearable sensors for smoking detection; (2) explore sensor modalities employed for detecting these manifestations; (3) evaluate underlying signal processing and pattern recognition methodologies and key performance metrics. The review identified five specific smoking manifestations targeted by sensors. The results of the review suggested that no system reached 100% accuracy in the detection or evaluation of smoking-related features. According to the authors, wearable devices require thorough testing under free-living conditions, as the testing of these reviewed sensors was mostly limited to laboratory settings.

The survey introduced in [6] focused on Arrowhead Framework in the Internet of Things and System of Systems dedicated architectures for smart cities and smart agriculture. According to the authors, the advantages of Arrowhead Framework technology are emphasized by analysis of several smart city use cases and a novel architecture for a telemetry system that will enable the use of Arrowhead technology in the smart agriculture area.

The survey introduced in [7] deals with the applications that come with the chest-worn Inertial Measurements Units (IMUs), analyzes 57 relevant studies and summarizes the existing methods, current challenges and future directions associated with them. The authors categorizes the works into seven applications: Seismocardiography, Activity Analysis, Posture Analysis, Localization, Voice Analysis, Swallow Analysis, and Context Retrieval. The authors discussed the inertial sensors used, as well as their placement on the body and their associated validation methods based on the application categories. Their investigations showed meaningful correlations among the studies within the same application categories. They also propose combining the discussed applications in a single platform, finding robust ways for artifact cancellation, and planning optimized sensing/processing architectures for them to be taken more seriously in future research.

The survey introduced in [8] systematizes knowledge in the field of industrial wearables’ safety to assess the relevance of their use in enterprises as the technology maintaining occupational safety, to correlate the benefits and costs of their implementation, and, by identifying research gaps, to outline promising directions for future work in this area. The authors categorized industrial wearable functions into four classes (monitoring, supporting, training, and tracking) and provide a classification of the metrics collected by wearables to better understand the potential role of wearable technology in preserving workplace safety. Furthermore, they discusses key communication technologies and localization techniques utilized in wearable-based work safety solutions. Finally, they analyzed the main challenges that need to be addressed to further enable and support the use of wearable devices for industrial work safety.

The systematic review introduced in [9] is focused on current positioning platforms for GNSS-denied scenarios. The authors have undertaken a comprehensive analysis of each component of the positioning and localization systems, including techniques, protocols, standards, and cloud services used in state-of-the-art deployments. Furthermore, the authors identified the limitations of existing solutions, outlining shortcomings in areas that are rarely subjected to scrutiny in existing reviews of indoor positioning, such as computing paradigms, privacy, and fault tolerance. The authors also examine contributions in the areas of efficient computation, interoperability, positioning, and localization. Finally, they provide a brief discussion concerning the challenges for cloud platforms based on GNSS-denied scenarios.

To sum up, this Special Issue has shown the potential applications of using wearable devices and smartphones as sensory devices and shed some light on the use and validation of wearables in different contexts/scenarios. However, a common evaluation setup involving wearable data is still a pending task of the research community. Having common evaluation metrics and data sets for the same topic may pave to way for comprehensive assessments of the proposed methods and research, not only enabling research reproducibility but also research collaborations between institutions.
